# Supporting women who develop poor postnatal mental health: what support do fathers receive to support their partner and their own mental health?

**DOI:** 10.1186/s12884-020-03043-2

**Published:** 2020-06-22

**Authors:** Andrew Mayers, Sarah Hambidge, Olivia Bryant, Emily Arden-Close

**Affiliations:** Department of Psychology, Bournemouth University, Poole, BH12 5BB UK

**Keywords:** Maternal mental health, Maternal postnatal depression, Fathers’ wellbeing, Maternity services, Pregnancy, Mental health

## Abstract

**Background:**

Research regarding support provided for poor maternal postnatal mental health (such as depression, anxiety disorders, and postpartum psychosis) is relatively common. Fathers appear to play an important role supporting partners but many feel alienated within maternity services. Research focusing on fathers is less common.

**Methods:**

The current qualitative study aimed to investigate fathers’ experience of support provided to fathers, to help support their partner should she experience poor postnatal mental health.

**Results:**

Twenty-five fathers participated in an online questionnaire regarding their experience of their partner’s poor postnatal mental health and the support provided to fathers to help her. Thematic analysis revealed three main themes and seven sub-themes. The themes were: ‘Support received to help support their partner’, ‘Support fathers wanted that was not received’ and ‘Father’s mental health’. The results highlight an overall lack of support for many fathers, despite many wanting support on how to help their partner, information on their own mental health and the services available. Fathers specifically wanted healthcare professionals to sign-post them to someone they can talk to for emotional support, and to be taught coping strategies which would help them to support both their partner and baby.

**Conclusions:**

The findings from this study suggest that health professionals and perinatal mental health services need a better understanding about what resources fathers need to support the mental health of themselves and their partner.

## Background

The arrival of a baby is often a joyous event. However, some women present with mental illnesses such as depression, anxiety disorders, and postpartum psychosis during pregnancy or post-birth [1]. Worldwide, mental health problems are experienced by 10% of pregnant women and 13% of women who have recently given birth [2], and are the biggest single preventable killer of women in the postpartum period; accounting for a quarter of maternal deaths, with one in seven of these from suicide [3].

The affected mother may struggle to interact with her baby in addition to experiencing physical and emotional changes. In some cases, this can negatively impact the child’s growth and development [4, 5]. Appropriate perinatal mental health support can provide a range of long-term benefits for the mother, the family including the baby, and wider society [6]. Support provided by healthcare professionals through the UK National Health Service (NHS) for maternal postnatal mental illness has improved in the last decade and can include self-help, psychological therapies, holistic approaches and specialist mother and baby units. However, inequalities in access to and experience of mental health services remain due to the lack of specialist perinatal mental health services [7]. The cost of the scarcity of such services to society is approximately £8.1 billion for each one-year cohort of births in the UK, with three-quarters of that relating to the impact on the child [8]. In recent years, NHS England has invested in extending these services, but data is not yet available regarding the impact of that investment.

A woman’s social network (i.e. their partner, family and friends) typically provides primary or additional support to the services offered by health care providers. Newly expecting mothers perceive their partner as their main support system [9]. Most new mothers live with their child’s father and perceive the father to have an important role in the support received [10]. Greater satisfaction with fathers’ involvement in caregiving was reported to help reduce postnatal depressive symptoms in mothers [11], while lack of support from the father can exacerbate mental health issues in new mothers [12]. This suggests that a supportive father can have a positive impact on maternal postnatal mental health. Furthermore, fathers hold an important role in the recognition of their partner’s mental health status. Specific risk factors, such as previous mental illness, can provide valuable guidance for early identification of when a mother’s mental health may be declining [13]. Supporting fathers’ knowledge of these risk factors, the presentation of symptoms and knowing where support can be sought may encourage quicker self-help seeking and engagement with interventions by the mother.

Although research suggests that fathers’ support likely benefits mothers, many men feel excluded by healthcare professionals during pregnancy and the postpartum period. Evidence suggests fathers feel alienated and are unclear regarding their role within maternity services [14]. Many fathers report feeling isolated and ignored during and following traumatic birth experiences. In our own research, we have shown that perceptions of trauma may occur following an emergency caesarean section or any other deviation from a birth plan, especially if there is potential risk of harm or death for the mother or child [15]. In Finland, Oommen et al. [16] found that in ‘mother and baby units’, many parents experienced difficulty adjusting to their new parental relationship and roles, but very little emotional support was provided. In some instances, fathers have felt supported during their partner’s pregnancy [15]. However, even in these instances, the support fathers received was perceived as minimal and did not last into the birthing experience or postnatally. This indicates a diminished recognition of the father’s role in supporting the mother’s mental health, particularly within maternity services and by healthcare professionals.

In a study by Nath et al. [17] it was found that both poorer maternal mental health and marital conflict contributed to poor mental health in fathers. Mothers and fathers’ mental health are highly correlated, and fathers’ risk of mental health problems during the perinatal period increases if their partner develops mental health problems [18], However, until very recently it was not even considered necessary to screen fathers for their mental health [19]. Fathers have confirmed their desire for greater recognition from healthcare professionals regarding the impact of postnatal depression on the family and feel that the ability to seek support during pregnancy and after the birth may relieve some of their stress [20]. These professionals may need to also consider how the father can help support their partner should she develop poor postnatal mental health. To include fathers within maternity services, and to help recognise the impact on their own mental health, men’s experiences must be explored. There are also needs to be more understanding about how fathers can support their partner for her mental health. Fathers desire more communication around their partner’s mental health than they currently receive [21]. Specifically, they desire information relating to their partner’s treatment and medication [22] to support their partner’s health and to help ease their own concerns and anxiety. Current literature lacks understanding of how fathers would like this information to be communicated.

The current study aimed to explore fathers’ experience of their partners’ postnatal mental health, including the impact on their own mental health during this period, and if any support provided to the fathers helped them support their partner.

## Methods

### Sample

Thirty-nine fathers accessed the online portal for the study. However, only 25 were used in the final analyses. Potential participants were excluded if they did not indicate consent to take part or if they did not fully complete the questionnaire.

#### Recruitment

Fathers were recruited voluntarily through the first author’s links with UK mental health charities and fathers’ support networks. Advertising was undertaken on social media platforms (such as Facebook and Twitter). Participants were eligible to be included if they were adult fathers (aged 18 or over), whose partner had experienced ‘poor postnatal mental health’. This was self-reported. No formal diagnosis was required as mothers often need support for poor mental health, regardless of the severity or diagnosis.

Demographic information is shown in Table 1. All of the fathers were living in the UK, were married or cohabiting at the time of study, and had been present at the birth of their child. No data were collected regarding ethnicity.

**Table 1:** Demographic information

Demographic information	Categories	Number of respondents
Partner’s report of poor mental health	Depression	15
	Anxiety	5
	Maternal OCD	2
	Psychosis	1
	Other	0
	Multiple	2
Location	Hampshire	5
	Oxfordshire	1
	Dorset	2
	Durham	1
	Surrey	1
	Scotland	2
	Wales	3
	Northern Ireland	1
	UK	1
	Unclear or misunderstood (i.e. at home)	5
	No response	3
Relationship with partner during the perinatal stage	Married	18
	Living together	7
	Separated	0
Father involvement during perinatal phase	Very involved	24
	Partially involved	1
	No involvement	0
Present in the birthing room	Yes	25
	No	0

### Procedure

Qualitative methods of data collection were chosen over quantitative analyses as these encourage a richer exploration of experiences [23]. A confidential online questionnaire was preferred over face-to-face discussions or interviews, as it was felt that anonymity would lead to greater willingness to participate. A new questionnaire was created for the purposes of this study (a copy of this questionnaire is available as supplementary file). It was developed with help from fathers’ mental health support groups with whom the first author works, to ensure that the questions were sensitive and appropriate. Recommendations were accommodated accordingly. It was distributed through Qualtrics®, an online portal. The questionnaire contained open questions which allowed for qualitative feedback regarding the father’s perspective of their experience and the support given to them. Questions regarding the father’s emotional wellbeing, and the support offered for this, were also included.

### Data analysis

Data were managed using NVivo 10 and analysed using thematic analysis [24] to allow detailed exploration of the responses. Responses were initially coded by one researcher (OB) to generate initial codes which were expanded, excluded or merged into overarching themes. Coding was an inductive and data-driven process, not informed by an a priori framework. The final themes were checked and verified by a second researcher (AM). Reflective notes were taken throughout analysis which allowed for complete transparency of the analytical process.

### Ethics

Informed consent, anonymity, confidentiality and the right of participants to leave the study at any time was preserved. Prior to completion of the study, participants were provided with an electronic information sheet and invited to confirm consent to take part. Upon completion, participants were provided with debriefing information. Anonymity was ensured through the removal of identifiers. All data were stored confidentially in accordance with UK General Data Protection Regulations.

## Results

**Fig. 1:**
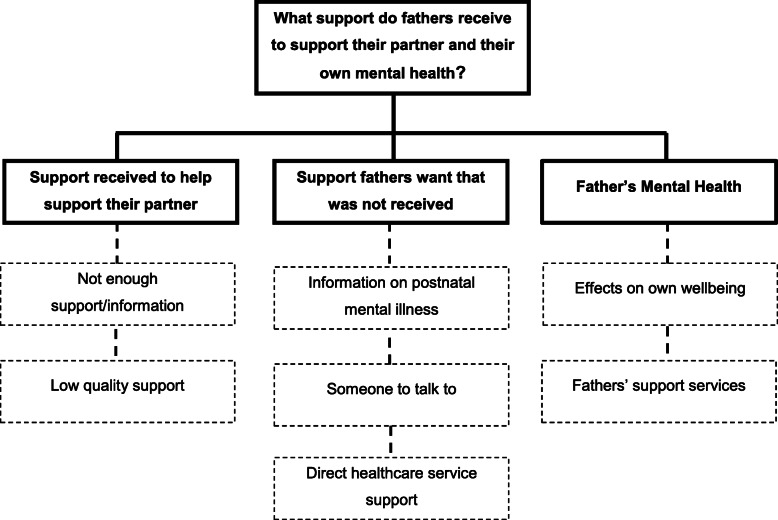
Themes and sub-themes identified

Data from 25 fathers were analysed. Thematic analyses identified 3 themes (see Fig. 1): “Support received to help support their partner” (Sub-themes: “Not enough support/information”, “Low quality support”); “Support fathers want that was not received” (Sub-themes: “Information on postnatal mental illness”, “Someone to talk to”, “Direct healthcare service support”) and “Father’s mental health” (Sub-themes: “Effect on own wellbeing”, “Fathers’ support services”).

### Theme 1: support received to help support their partner

This theme examined what help was provided to fathers to enable them to better support their partner through poor postnatal mental health. This support also includes maternal and mental health services directed at the mother, and the mother and father’s experience of interaction with these services from the fathers’ perspective. This main theme contained two sub-themes, “Not enough support/information” and Low-quality support".

#### Sub-theme 1: not enough support/information

Twenty participants reported not receiving any support or information regarding postnatal mental illness and how they could support their partner pre- or post-birth. The two participants who received information considered it inadequate. The remaining participants believed they received enough support after the birth of their child, whilst another suggested that they did not need any support post-birth. The lack of support and information provided often left participants feeling confused and/or frustrated regarding how to help their partner, with some admitting it negatively affected their ability to support the mother.

*“I didn’t know how to help her.”* (P1).

Many participants, mainly those who did not receive any support, would have welcomed any type of support or information from healthcare professionals. Participants felt being asked if they required help or support would have been beneficial both pre and post birth.

*“Any offer of help and support would be useful*.” (P2).

#### Sub-theme 2: low quality support

Twelve of the participants who received support or information claimed the support provided by healthcare professionals was typically readable information (i.e. leaflets) and/or very brief verbal signposting to videos or other resources. For four participants this support was second-hand; information was narrated by partners to the participants. Only a very small amount of this information focused on postnatal mental illness.

*“I read the information my wife was given.”* (P3).

No participants reported receiving good quality support directed specifically towards them. Participants felt the lack of support suggested a reduced acknowledgement of the father’s role in the mother’s care.

*"I received support but it was all so fast paced, it [information]*

*didn’t cover anything about the father and I felt lost."* (P4).

Twelve participants recalled poor information and/or unprofessional services within the healthcare setting, which contributed further to their sense of inadequate support being provided.

*“[The] crisis team were useless, trying to call them after giving us a wrong number”* (P5).

Those whose partner and baby were admitted to a mother and baby unit blamed poor healthcare facilities for limiting the amount of time they could support their partner following the birth. This created a stressful environment and situation for both parents.

*“[I] had to go home as facilities wasn’t there for me to stay”* (P6).

In several cases, the perceived lack of support and information led both parents to seek help elsewhere.

*“[We are] now saving in order to get private therapy”* (P3).

### Theme 2: support fathers want that was not received

This second theme covered what type of support fathers would like to have received before and after the birth. This theme contains three sub-themes, “Information on postnatal mental illness”, “Someone to talk to” and “Direct healthcare service support”.

#### Sub-theme 1: information on postnatal mental illness

Eighteen of the participants would like to have received more support and information both prior to and after the birth of their child. They felt that they would have benefited from knowing warning signs for poorer postnatal mental health and ways to help their partner cope.

*“[To be] made aware of symptoms, and it would have been good to know what to do when you suspected it [mental health problems].”* (P7).

These eighteen participants suggested information on their partner’s specific mental health condition would have supported them in understanding the condition, the symptoms and how to best help their partner. They felt this information would be best presented in the form of easy to access leaflets or other written materials available on the internet because these types of resources can be easily accessed at any time point. Two participants expressed concern about forgetting information they were verbally told about their partners condition by health care professionals, especially if the information was narrated during a stressful time (i.e., a partner having a psychotic episode).

*“A basic understanding of depression and how to help [when] dealing with psychosis episodes … Leaflets on what to look out for, as you can’t always remember what you are told in the immediate aftermath”* (P6)

#### Sub-theme 2: someone to talk to

Seven participants stated they would have benefitted from having someone to talk to about their situation. They felt it would have helped them to understand what was happening to their partner, what the options might be regarding their partner’s treatment plan, and how they could support their partner. These participants would have liked their partner’s treatment and recovery plan explained by a healthcare professional.

*“A specialist to sit with me and explain the situation and care plan.”* (P4).

Participants viewed having someone to talk to as a useful form of emotional support. Many felt they were given little to no emotional support, such as having someone to talk to, to cope with their own mental health issues.

#### Sub-theme 3: direct healthcare service support

Twelve participants said they would have appreciated more engagement and communication from maternal healthcare providers, in addition to increased access to mental healthcare professionals for their partner. They believed that better guidance on their partners diagnosis and treatment could be obtained from a range of healthcare professionals.

*“Some Mental Health support, as well as social worker support and referral to a therapist”* (P8).

Two participants suggested that mothers should be able to access services (i.e. counselling or talking therapy) where they could speak to a healthcare professional on their own without any family members or other healthcare professionals being present. These fathers felt that their partners might have been more likely to speak openly about their experiences when seen alone.

*“we could have both seen the same therapist but individually and then together.”* (P9).

When discussing their own support needs, all twelve participants stated that they would have benefitted from training in coping strategies to help them both support their partner and care for their own mental health:

*“Having coping strategies and understanding how to keep calm!”* (P5).

### Theme 3: fathers’ mental health

The final theme explores the participants’ mental health in light of their partner’s symptoms including any support they personally received from healthcare professionals and outside organisations. This theme has two sub-themes, “Effect on own wellbeing” and “Fathers’ support services”.

#### Sub-theme 1: effect on own wellbeing

Twenty-three fathers believed that some aspect of their overall wellbeing had been directly affected by their partner’s mental health during the perinatal period. This often led to feelings of low mood, anxiety and general stress and affected physical areas of their life such as their ability to sleep, concentrate and even care for their child.

*"I was scared. I c*ould *not sleep. My memory lapsed and I cried too often.*

*Made me feel like I couldn’t be as supporting to my son"* (P10).

Four participants stated that these heightened physical changes and emotional responses had a negative impact on their relationship with their partner resulting in arguments, spending time apart and a decline in the support they offered each other:

*“Things became very difficult and pushed us apart.” (P7).*

Nevertheless, two participants confirmed that although the postnatal period was a stressful time, they were able to cope with the emotional demands, suggesting that they were either more resilient to the emotional effects of their partner’s poor mental health, or less willing to admit vulnerability. Several of the participants felt they needed to be seen to remain emotionally and mentally strong to support their partner and baby, despite coping with their own mental health.

*"It was challenging supporting my partner and baby and managing with*

*my own mental health, but I coped"* (P11).

#### Sub-theme 2: fathers’ support services

All twenty-five participants reported they rarely received support for their own mental health, and any support received was minimal. This lack of support from healthcare professionals led to fathers experiencing feelings of isolation and confusion around their own mental health issues.

*“My wellbeing was of little interest to midwifes, health visitors … [I] had not given birth so had no cause for sympathy. A leaflet for my wife and a page for the fathers to read which wasn’t enough”* (P10).

Participants agreed there was not enough information (and reassurance) on father-child bonding activities, something which they worried about, leaving new fathers feeling they were forgotten or treated with little sympathy.

*“There was no information … .. how to understand that it could take a while for your child to bond as it does with the mothers”* (P10).

Consequently, nine participants felt there was an extreme imbalance between the level of support fathers receive from healthcare professionals compared to mothers.

*“Mothers have support from midwives and health visitors, but dads get nothing”* (P12).

Although these nine participants acknowledged that the focus should primarily be on the woman, as she carries the baby and gives birth to their child, they still felt fathers should be offered more information and support than that currently made available by healthcare professionals.

“*I understand the focus was and should be on my partner, but a bit of concern … would have been most welcomed.”* (P11).

Any “better-quality” support participants received for their mental health problems was typically provided by organisations outside of the major healthcare services. Participants felt high quality services directed towards fathers’ mental health within current healthcare providers was currently inadequate but were able to access support from external organisations and groups.

*“I’m now getting [the] support that I need as I did meet with a fathers group where Mark was present, and what a great help he was”* (P13).

## Discussion

The current study explored fathers’ experience of their partner’s poor postnatal mental health and the resources provided to them to help support their partner. Fathers are at risk of mental health problems during this timeframe, expressly if their partner develops mental health problems [17]. The current study also supported previous findings that showed fathers require better recognition of their mental health needs from healthcare professionals [20]. Historically, it has not been considered necessary that fathers should be screened to determine if they are experiencing symptoms of a mental health condition [19]. More recently, NHS England announced plans to screen fathers for their mental health (at least in parts of the UK), but precisely what that will mean in practice is still not known.

Many fathers spoke of the negative impact their partner’s mental health had on both their emotional and physical wellbeing. Fathers received little to no support yet desired specific types of support to be able to help their partner, as well as themselves. Similarly, Darwin et al. [14] found that fathers felt isolated within maternal services and confused regarding their role. However, Darwin et al. [14] stated that most fathers felt that the focus should be on their partner, whereas within the current study, many fathers felt frustrated that they had not been included in the support and information provided. This difference may be due to the current study focusing on maternal postnatal mental health rather than maternity services overall. Poor maternal postnatal mental health may have led some fathers to believe that they should take a more active role in their partner’s care due to the more challenging circumstances.

Participants highlighted that support received was often insufficient to help with what followed. This mirrored our findings from Daniels et al. [15] in which fathers felt that support was minimal and not provided throughout the perinatal experience. Although that research was related to birth trauma, and not postnatal mental illness, both studies recognise specific areas within support services that may be lacking during a very stressful time for fathers, such as informational support and aftercare.

Many fathers would have liked an offer of support. This suggests a distinct lack of support currently offered to fathers. Similarly, in our study of fathers’ experiences of birth trauma [15] we also found that many fathers would have liked a lot more general support, ranging from simply being acknowledged to written information which can be referred to later.

Fathers desired more information specific to postnatal mental illness, such as warning signs and potential symptoms, and information on how best to help their partner through this difficult time. Similar findings were reported by Reid et al. [22]. This qualitative study identified the experiences of 17 fathers when their partners were admitted with their infants to a psychiatric mother and baby unit. The study identified that fathers’ desire information relating to their partner’s treatment and medication and recommended the provision of an information pack and regular one-to-one meetings between fathers and healthcare staff [22].

Many fathers highlighted that they would have liked improved communication with health professionals regarding their partner’s condition to help them understand the situation. This finding was confirmed in a qualitative study by Marrs et al. [21], which explored eight fathers’ paternal roles and relationships when their partner and baby were admitted to a perinatal mental health unit. Fathers also felt they would have benefitted from having someone to talk to regarding their emotional needs, in line with research by Letourneau et al. [20], where fathers stated that they would have benefitted from having someone to talk to.

Fathers wanted more interaction with, and involvement from, healthcare professionals particularly with specialists in mental health. This was primarily in relation to their partner’s need for greater service involvement and access to professional help. Similarly, Higgins et al. [25] highlight the lack of maternal mental health specialists in the Republic of Ireland. Lack of specialist services can, in turn lead to reduced service guidance for individuals with postnatal mental illness and their loved ones. Although Higgins et al. [25] explored the mother’s personal experience, rather than the father’s, both studies highlight the lack of access to specific mental health services.

Poor maternal postnatal mental health appears to have a knock-on effect on many fathers’ psychological and physical wellbeing. In support of this idea, research conducted by Nath et al. [17] highlights how higher levels of depressive symptomology in new fathers appeared to be linked to postnatal mental illness within the mother. Early interventions to help fathers cope with stress may be needed in order to reduce the risk of future deterioration of emotional wellbeing. Further, in line with Nath et al. [17], fathers felt marital conflict was linked to their depressive symptomology. However, Nath et al. [17] suggested that this marital conflict was a risk factor for depressive symptomology, whereas the current study appears to suggest the opposite.

There was a distinct lack of service support and general recognition for father’s mental health. Many fathers felt little sympathy was provided to them. This mirrored Letourneau et al. [20] in which fathers desired a greater recognition of the impact of maternal mental illness on the family as well as awareness of paternal postnatal mental health. The current study also found that this lack of support targeted towards fathers was more prominent within healthcare service providers. This was also mirrored in our birth trauma research [15] in which many fathers felt they received minimal personal support following the birth. However, the current study did show an offer of support from some external organisations.

### Implications for research and clinical practice

One of the key outcomes from this study indicated that fathers feel that they are not getting enough recognition from healthcare professionals about how they might play an active role in supporting their partner’s mental health. This indicates that fathers need to be better informed about how they can help their partner, including signposting to the support services that are now beginning to develop across the UK (an overview of some of these is updated frequently at http://www.andrewmayers.info/fathers-mental-health.html).

It is also clear that maternal mental health has an impact on the paternal mental health. Without recognition, these symptoms may be missed. The ability to seek support throughout the perinatal period may relieve fathers of some of the stress and overwhelming feelings they may be experiencing. Awareness of both partners’ needs to be embedded into perinatal healthcare training for all professional staff. Information and support for fathers may be especially important when they have been exposed to more extreme circumstances, including witnessing a traumatic birth where the life or wellbeing of his partner and/or baby were threatened.

Several of the findings from this study highlighted that fathers perceived a lack of healthcare education and training regarding their needs, and that there are too few specialist support services. Many fathers in this study reflected that earlier intervention may have helped reduce the impact on their mental health in the longer term. Fathers felt that more focus should be paid to increasing sensitivity and support offered within the NHS and other national local healthcare providers. These needs are beginning to be addressed in the UK.

In November 2018, NHS England announced that, where mothers are referred to perinatal mental health services, fathers will now also be screened for their mental health. This the first time that fathers have been considered, and it is a major step forward, but it could be argued that there is still some way to go to fully address the evidence raised in the current study. While the proposals recognise the evidence regarding how maternal wellbeing impacts on the father, not all mothers with poorer mental health meet the criteria for full referral, meaning fathers might still be missed.

More recent proposals in the NHS Long-Term plan indicate that future perinatal mental health services could draw on wider community support, rather than rely wholly on NHS commissioned services. This could potentially identify more fathers in need of support, and facilitate more localised support groups, but how this will be done is still not clear. Ideally, this could be undertaken through statutory health provision alongside third sector and local charity services. Further recent developments have shown that many perinatal health professionals, including midwives and health visitors, are now additionally being trained and educated regarding the needs of fathers at this time.

In this study fathers indicated that they felt their poor mental health contributed to marital conflict. This contrasted with previous findings by Nath et al. [17] that it was the partner conflict that led to poorer mental health. Further research could be undertaken to explore this in more detail. Additional research is also needed to explore healthcare professionals’ perceptions of what training and education they need to support fathers’ mental health more effectively. The lead author is currently undertaking some of that research.

### Limitations

The use of an online qualitative questionnaire was chosen in order to respect participant anonymity. While such methods allow a richer investigation of perception than might be achieved from quantitative scales, there was not an opportunity to develop those perceptions in more depth. Future studies might consider employing interviews or online discussion, while maintaining the assurance of confidentiality. These methods might allow the researcher to explore specific aspects of the participant’s response.

Data regarding ethnicity was not collected. Such information would have enabled identification of whether lack of support received by fathers is a particular issue for specific ethnic groups. While there is some evidence about maternal mental health according to ethnicity e.g. [26], similar research for fathers is lacking.

## Conclusion

The outcomes from the current study indicate that health professionals and perinatal mental health services need a better understanding about what support fathers feel they may need at this time. Most fathers in this study perceived informational support to be most valuable. Therefore, further information and guidance for fathers regarding postnatal mental health could be implemented throughout the perinatal period to aid their ability to recognise and support their partner’s mental wellbeing. This, in turn, could potentially lead to quicker help-seeking and recovery. In addition, as many fathers noticed a need for greater wellbeing support, increased access to support for both partners could potentially improve overall wellbeing within the family. This could be delivered through holistic services within the community that represent a combination of statutory health provision alongside third sector and charities.

## Supplementary information

**Additional file 1.** Online questionnaire.

## Data Availability

In order to maintain participant anonymity full questionnaire transcripts cannot be made available.
