# Supplementary material for: Supporting women who develop poor postnatal mental health: what support do fathers receive to support their partner and their own mental health?
Source: BMC Pregnancy Childbirth. 2020 Jun 22;20:359. doi: 10.1186/s12884-020-03043-2 (PMC7310127; doi:10.1186/s12884-020-03043-2)
Supplement: Supplementary file 1 — Additional file 1. Online questionnaire. [file 12884_2020_3043_MOESM1_ESM.docx]

**Demographics**

Q1 – Where did you live during the perinatal stage?

Q2 – What postnatal mental illness did/does your partner suffer from?

- Postnatal depression
- Postnatal anxiety
- Postnatal psychosis
- Maternal OCD
- Other (or state any undiagnosed condition)

Q3 – In relation to the person who suffered from postnatal mental illness, were you:

-Married

-Living together

-Partners but not living together

-Separated

**Fathers involvement**

Q4 – How involved were you with your partner’s pregnancy? (please specify type of involvement i.e. attending antenatal appointments, scans, etc)

Q5 – Were you present in the birthing room?

Q6 – How involved were you with your partner following the birth?

**Support before the birth**

Q7 - Prior to the birth did you have any previous knowledge or experience of mental health problems?

Q8 - Did you receive any support or information relating to postnatal mental illness prior to the birth? (if yes please specify)

Q9 - In hindsight do you feel that you were given enough support regarding maternal postnatal mental illness at this stage?

Q10 - What support or information would you have liked to have at this stage?

**After birth**

Q11 - Did you receive any support or information on postnatal mental illness following the birth? (if Yes please specify)

Q12 - Do you feel that you were given enough support regarding maternal postnatal mental illness following the birth?

Q13 - What support or information would you have liked to have had at this stage?

Q14 - How did your partner/wife’s illness affect your own mental health?

Q15 - What support were you given in regards to your own wellbeing? (please specify what type of support and where this was from)

Q16 - Is there any additional information you would like to share with us that has not been covered?
